# Washout allometric reference method (WARM) for parametric analysis of [^11^C]PIB in human brains

**DOI:** 10.3389/fnagi.2013.00045

**Published:** 2013-11-27

**Authors:** Anders Rodell, Joel Aanerud, Hans Braendgaard, Albert Gjedde

**Affiliations:** ^1^Department of Nuclear Medicine and PET Centre, Aarhus University HospitalAarhus, Denmark; ^2^Department of Neurology, Aarhus University HospitalAarhus, Denmark; ^3^Department of Neuroscience and Pharmacology, University of CopenhagenCopenhagen, Denmark

**Keywords:** Alzheimer's disease, CBF, Aβ, PIB, flow normalization, parametric imaging

## Abstract

Rapid clearance and disappearance of a tracer from the circulation challenges the determination of the tracer's binding potentials in brain (*BP*_ND_) by positron emission tomography (PET). This is the case for the analysis of the binding of radiolabeled [^11^C]Pittsburgh Compound B ([^11^C]PIB) to amyloid-β (Aβ) plaques in brain of patients with Alzheimer's disease (AD). To resolve the issue of rapid clearance from the circulation, we here introduce the flow-independent Washout Allometric Reference Method (WARM) for the analysis of washout and binding of [^11^C]PIB in two groups of human subjects, healthy aged control subjects (HC), and patients suffering from AD, and we compare the results to the outcome of two conventional analysis methods. We also use the rapid initial clearance to obtain a surrogate measure of the rate of cerebral blood flow (CBF), as well as a method of identifying a suitable reference region directly from the [^11^C]PIB signal. The difference of average absolute CBF values between the AD and HC groups was highly significant (*P* < 0.003). The CBF measures were not significantly different between the groups when normalized to cerebellar gray matter flow. Thus, when flow differences confound conventional measures of [^11^C]PIB binding, the separate estimates of CBF and *BP*_ND_ provide additional information about possible AD. The results demonstrate the importance of data-driven estimation of CBF and *BP*_ND_, as well as reference region detection from the [^11^C]PIB signal. We conclude that the WARM method yields stable measures of *BP*_ND_ with relative ease, using only integration for noise reduction and no model regression. The method accounts for relative flow differences in the brain tissue and yields a calibrated measure of absolute CBF directly from the [^11^C]PIB signal. Compared to conventional methods, WARM optimizes the Aβ plaque load discrimination between patients with AD and healthy controls (*P* = 0.009).

## Introduction

The marker [^11^C]PIB is a radiotracer of amyloid-β (Aβ) plaque load, used clinically to diagnose Alzheimer's disease (AD) (Cohen et al., 2012). Despite its wide use, several challenges face the actual quantification of this and other Aβ probes (Ducharme et al., 2013), as recently reviewed by Kepe et al. (2013). Unfortunately, no dose-escalation and competitive displacement studies have been performed for any Aβ imaging agent. Thus, the *in vivo* binding properties of these tracers have not been fully characterized (Villemagne et al., 2012), including the differences of binding to specific tissue types (Fodero-Tavoletti et al., 2009) such as estrogen-related receptors. Functionally, AD syndromes are associated with degeneration of specific functional networks, and amyloid deposition as measured with [^11^C]PIB explains at most a small amount of the clinico-anatomic heterogeneity in AD (Lehmann et al., 2013). Even if [^11^C]PIB binds uniquely to extracellular Aβ plaque formations, the relevance of such binding may be coupled to autophagy as recent evidence suggests that Aβ secretion and plaque formation depend on autophagy (Nilsson et al., 2013). Given its widespread clinical use, enigmatic binding properties, and the relative high cost of positron emission tomographic procedures, better methods for the quantification of this tracer in relation to other clinical parameters adds to the clinical relevance of the tracer.

The rapid initial clearance from the circulation suggests that [^11^C]PIB is subject to flow-limited uptake, such that the parametric analysis of the binding must take blood flow and washout kinetics into account. There is therefore a need to couple Aβ measures to both metabolic function and CBF deficits (Johannsen et al., 2000), as well as the loss of flow variability observed in AD (Rodell et al., 2012).

Three methodological problems complicate the correct quantification of binding of flow limited and rapidly metabolising tracers like [^11^C]PIB in human brain (Møller et al., 2009).

The first is the rapid disappearance of the tracer from the circulation and the consequent brief exchange with brain tissue. Conventional binding studies by equilibrium methods are affected by this absence of a continuing source of tracer in the circulation. Reference and binding regions independently clear tracer from the respective volumes of distribution, and the tracer in the reference region no longer is a proper surrogate for the tracer in the circulation. The time-activity functions of different regions now depend on regional properties of binding, blood flow, and blood-brain barrier permeability, rather than on a common source of tracer in the circulation.

The second is the uncertain choice of a region of reference of specific flow and no specific binding. The cerebellum is held to be little affected by amyloid deposition in AD, and the cerebellar gray matter often serves as reference region for measures of [^11^C]PIB retention (Landau et al., 2013), but other regions have been considered as well, including whole cerebellum (Joshi et al., 2012), and the Pons (Klunk et al., 2007; Knight et al., 2011).

The third potential pitfall is the influence of cerebral blood flow and blood-brain barrier permeability differences on regionally specific binding of [^11^C]PIB. If the tracer clears from multiple compartments in a single region of interest with different quantities of exchangeable and bound tracer, it is possible that both flow and permeability differences can mimic or mask changes of binding.

The problems facing conventional [^11^C]PIB quantification are manifest when [^11^C]PIB retention is evaluated in healthy subjects and patients with AD with different degrees of Aβ deposition. For example, it has been reported that PIB may bind differentially to polymorphic Aβ aggregates in some humans as well as in animals (Rosen et al., 2010; Ikonomovic et al., 2012). Additionally, apparent retention of PIB is evident in cerebral white matter both *in vivo* by PET (Fodero-Tavoletti et al., 2009) and *in vitro* by postmortem auto-radiography (Klunk et al., 2004; Svedberg et al., 2009).

In the present study we aimed to establish a method of global parametric mapping of the binding potential (*BP*_ND_) of [^11^C]PIB that would take the particular kinetic properties of [^11^C]PIB exchange with brain tissue into account. For evaluation of these properties we compared tree non-invasive assays of [^11^C]PIB binding in brain of healthy subjects and a group of patients suffering from AD.

First, we applied a new model of tracer clearance, Washout allometric reference method (WARM), which we designed to map the washout of the tracer from regions of specific and non-specific binding. Previous approaches to the analysis of wash-out of tracers included the early “Height-Over-Area” method of Zierler (1965) and the recent “Hypotime” method of Møller et al. (2009), from both of which the current approach borrows, taking into account the methodological weaknesses discussed by Kanno and Uemura (1975).

Second, measures of regional tracer clearances were used to identify a reference region of negligible specific binding.

Third, the simplified reference region method (SRTM) (Lammertsma and Hume, 1996), also accounts for flow-dependent differences in tracer delivery, but contrary to the WARM method it assumes that differential equations modeling the radioactivity in a region of interest and a reference region are continuously coupled by exchange with a well defined arterial contribution of tracer (Lammertsma and Hume, 1996).

Fourth, the reference region subsequently served to obtain parametric maps of *BP*_ND_ by means of a well established and clinically popular and simple ratio measure (SUVR) of the area under the specific retention curve (AUC), at a presumed optimal time range 40–60 min after i.v. administration of the tracer, relative to the AUC of the retention in the reference region.

Altogether, we evaluated the methods that are used to establish binding potentials, as well as the effect of flow-dependent correction on the binding potential of [^11^C]PIB.

## Methods

### Subjects

Six patients with AD (four women and two men) with an average age of 65 (*SD* = 7) years and moderately reduced Mini-Mental State Examination (MMSE) scores of 22–25 volunteered to complete the tomography. The patients were recruited from the local Dementia Clinic and screened by an experienced neurologist to fulfill the criteria for probable Alzheimer s disease.

Eight healthy age-matched HC volunteers with a mean age of 68 (*SD* = 5) recruited by public advertisement served as controls. They all had a normal physical and neurological examination and had a MMSE between 28 and 30. To exclude cognitive impairment they were furthermore examined with the Danish version of CAMCOG (Lolk et al., 2000).

We obtained written informed consent from all subjects to the protocols approved by the Regional Science Ethics Committee in accordance with the Declarations of Helsinki. We previously reported some PET results from the same subjects (Rodell et al., 2012; Gjedde et al., 2013).

### Positron emission tomography

#### Image acquisition

All subjects had positron emission recordings, one or two with [^15^O]water and one with [^11^C]PIB, in the 3D mode of the ECAT High Resolution Research Tomograph (HRRT, CTI/Siemens, Knoxville, TN, USA) in a quiet room with the subjects resting in a supine position with eyes open. One of the male AD patients only completed the [^11^C]PIB recording. The images were reconstructed with 3D-OP-OSEM point spread function reconstruction (Varrone et al., 2009) using 10 iterations and 16 subsets with FWHM at approximately 1.5 mm. The reconstructed images were corrected for random and scatter events, detector efficiency variations, and dead time. Tissue attenuation scans were performed using a rotating 68Ge source. Dynamic emission recordings lasting 3 min (21 frames) were initiated upon bolus intravenous injection of [^15^O]water (500 MBq) or injection of [^11^C]PIB (500 MBq). Catheters (Artflon and Venflon, Becton Dickinson, Swindon, UK) were inserted in the right radial artery and left cubital vein and arterial blood radioactivity was measured every half second for the duration of the PET scan by an automated blood sampling system (Allogg AB, Mariefred, Sweden), cross-calibrated with the tomograph, and then corrected for external delay and dispersion. For anatomical orientation, high-resolution T1-weighted MR images were obtained at 1.5 or 3 T (GE Sigma Systems).

#### Image registration and segmentation

The summed emission recordings of [^15^O]water and [^11^C]PIB were automatically co-registered to the individual MRI scans using a six parameter affine transformation. Individual MR Images were co-registered to a locally generated version of the common stereotactic space (ICBM, Montreal Neurologic Institute) (Mazziotta et al., 2001) using a combination of linear and non-linear registrations (Collins et al., 1994; Grabner et al., 2006). After the calculation of the final 16 mm non-linear PET-Talairach transformation grid, dynamic emission recordings were re-sampled into common coordinates. Regional *BP*_ND_, R_1_, and CBF measures were obtained from parametric PET image maps using standard model based segmentation (Collins et al., 1994; Grabner et al., 2006). The regions analyzed were cerebral cortex excluding cerebellum (CORT), putamen (PU), caudate nucleus (CN) frontal (FL), occipital (OL), parietal (PL), and temporal (TL) lobes, as well as white matter (WM), and the cerebellar gray matter (CERB).

### Quantification of [^11^C]PIB retention

#### Reference region ratio measure (SUVR)

The [^11^C]PIB retention can be calculated by determining the accumulation relative to a reference tissue to obtain a ratio measure (SUVR). The ratio measure is the fraction of the region-of-interest integral of [^11^C]PIB accumulation at steady-state, assumed to have been established no later than this time after injection (*t*_*s*_ = 40 min), extended to the end (*t*_*e*_ = 60 min), relative to the integral of the [^11^C]PIB accumulation observed in the same period in the reference region. In the reference region, we assume the accumulated tracer as function of time, *m*_ND_(*t*) to represent non-specific binding after delivery of the tracer by homogeneous flow to all voxels of the reference region. In this context, we further assumed the interval from *t*_*s*_ = 40 min to *t*_*e*_ = 60 min to be sufficient to establish steady-state or secular equilibrium in all regions, as the basis for the definition of the volume of distribution of the tracer, *V*_T_, as the sum of the volumes of distribution of non-displaceable tracer (*V*_ND_) and an additional volume of distribution of displaceably bound tracer.

#### Washout allometric reference method (WARM)

In the case of negligible input from the circulation after the initial brief uptake, the tissue time-activity curves of the radio ligand are established by the radioactivity initially persed to the tissue and the subsequent washout from the brain regions of uptake. The WARM method specifically takes this condition into account and uses only the differences among washout rates from regions with different properties of binding, blood flow rates, and blood-brain barrier permeability. The condition means that the differential Equations (1) and (2)

(1) $$\frac{\text{d}m^{*}(t)}{\text{d}t}=K_{1}c_{a}(t)-k_{2a}m^{*}(t)$$

and

(2) $$\frac{\text{d}m_{\text{ND}}^{*}(t)}{\text{d}t}=K_{1}^{\text{ND}}c_{a}(t)-k_{2}^{\text{ND}}m_{\text{ND}}^{*}(t)$$

are linked only while the tracer is dispersed from well-defined *c*_*a*_, i.e., during the brief uptake period until maximum peak (within 2–10 min timeframe) when washout is assumed to be negligible.

Equations (1) and (2) also form the base of the two compartment Simplified Reference Tissue Model (SRTM) (Lammertsma and Hume, 1996). The term *K*_1_ is the unidirectional clearance of the tracer *c*_*a*_ by the tissue, *K*^ND^_1_ is the clearance of the tracer *c*_*a*_ by the reference region, m^*^ and m^*^_ND_ are the measured PET signal in the tissue (with displaceable binding) and reference, respectively. The term *k*_2*a*_ defines the apparent measurable washout rate constant for the ROI. The term *k*_2_ is the unknown washout rate for non-specifically bound tracer of the same region of interest, and *k*^ND^_2_ defines the measurable washout rate of non-specifically bound tracer in the reference tissue into the plasma. The uncoupling of the first and the second term on the right hand side of the equations means that elimination of the first *K*_1_ and *K*^ND^_1_ terms yields the equations for the washout part of the signal.

(3) $$\frac{\text{d}m^{*}(\text{t})}{\text{dt}}=-k_{2a}m^{*}(t)$$

(4) $$\frac{\text{d}m_{\text{ND}}^{*}(\text{t})}{\text{dt}}=-k_{2}^{\text{ND}}m_{\text{ND}}^{*}(t)$$

The rate constants *k*_2_ and *k*^ND^_2_ of washout of non-specifically bound tracer from tissue to plasma are linked by *R*_1_,

(5) $$k_{2}=R_{1}k_{2}^{\text{ND}}$$

The distribution volume ratio(DVR) can be expressed as an allometric relationship between the logarithmic of the fraction of remaining tracer in a ROI relative to the deposited amount before washout, and the reference region where $\left(\frac{\text{DVR}}{R_{1}}\right)$ is the scaling exponent, found in this formulation by log–log linearization as,

(6) $$\ln\left(\frac{m_{\text{ND}}^{*}(t)}{m_{\text{ND}}^{*}(0)}\right)=\left(\frac{\text{DVR}}{R_{1}}\right)\ln\left(\frac{m^{*}(t)}{m^{*}(0)}\right)$$

where the ratio m^*^(*t*)/m^*^(0) is defined by the amount of tracer m^*^(*t*) remaining relative to the amount of tracer m^*^(0) initially deposited before the washout. Similarly for m^*^_ND_(*t*)/m^*^_ND_(0).

For direct calculation without linearization, *BP*_ND_ can be found using the operational equation

(7) $$BP_{\text{ND}}(T)=\frac{m^{*}(0)\int_{0}^{T}\left(\ln\left(m_{\text{ND}}^{*}(t)\right)-\ln\left(m_{\text{ND}}^{*}(0)\right)\right)\text{d}t}{m_{\text{ND}}^{*}(0)\int_{0}^{T}\left(\ln\left(m^{*}(t)\right)-\ln\left(m^{*}(0)\right)\right)\text{d}t}-1$$

Intuitively, when log transformed, the fraction part (i.e., DVR) of this equation states that the nominator is the accumulated log-signal for the reference tissue relative to how much was present before washout, this difference is scaled by the initial tracer amount of the ROI. The denominator describes the accumulated log-signal for a ROI or voxel relative to how much was present before washout. This difference is scaled by the start amount of the reference region. Thus the fraction is corrected both for flow, i.e., initially deposited tracer, and the exponential behavior of the washout. For a more detailed derivation please refer to the Theory section.

**Figure 1:**
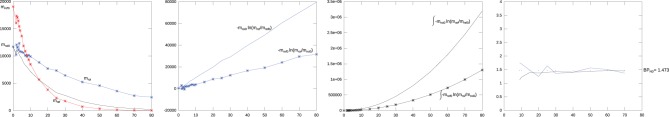
**Behavior of the parts of the warm method**. The first leftmost panel shows two initial simulated mono-exponential washout curves, a reference curve (*k*^ND^_2_ = 0.92) in red, and a binding curve (*k*_2*a*_ = 0.98) in blue. The black curve shows the flow normalized version of the reference curve normalized to the ROI curve, with 20% Gaussian noise added. The second panel shows the denominators and nominators from Equation (40) as function of time, i.e., the fraction between these two ideally linear factors form the binding potential. The third panel shows the similar integrated nominator and denominator from Equation (43). The integration stabilizes the result. The fourth panel shows the *PB*_ND_ from Equation (40) in blue, and from Equation (43) in black. The dotted line is the theoretical true value for *PB*_ND_. The *PB*_ND_ from both Equations (40) and (43) converge toward the true value with increasing tomographic duration.

Figure 1 illustrates the dynamics of the nominator, the denominator, and the *BP*_ND_(*T*) terms of Equations (43) and (40) (in the Theory section) for simulated ROI and reference curves with 20% Gaussian noise added. As seen, the nominator and the denominator from Equation (40) (in panel 2 from the left) are stabilized by the integration in Equation (43) (panel 3 from the left). Panel 4 illustrates the convergence of the *BP*_ND_(*T*) estimates toward the theoretical result.

#### Simplified reference tissue method

The simplified reference tissue method (SRTM) (Lammertsma and Hume, 1996) yields binding potential when a single tissue compartment model fits the data. SRTM solves differential equations similar to (25) and (26) (Equations (1) and (5) in Lammertsma and Hume (1996)). The method applies standard non-linear regression analysis to establish the relationship between the tissue concentrations of the region of interest and a reference region tissue such as typically the cerebellar gray matter in the case of [^11^C]PIB. The parameters estimated are *R*1, *k*_2_, and *BP*_ND_. *R*1 accounts for differences of delivery to the regions of interest and reference.

#### Flow dependence of specific binding measure

**Figure 2:**
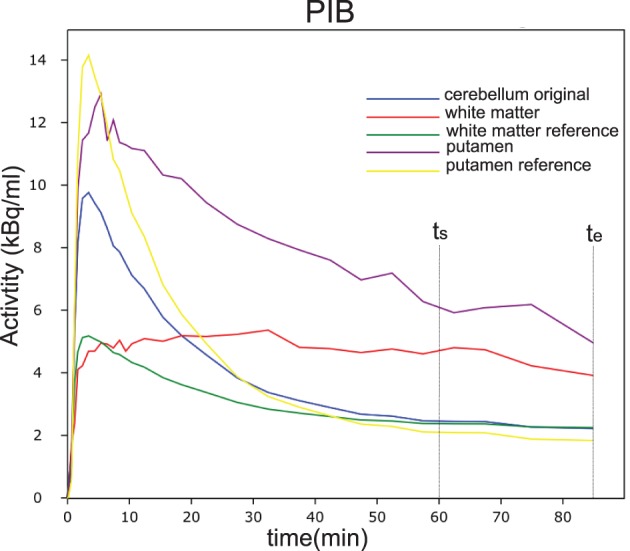
**Time activity curves of PIB from a single subject in different regions of interest (ROI)**. In blue is shown the measured cerebellar reference curve. The purple and yellow curves are the putamen curves as well as the estimated reference curve. The red and green curves are the curves for a small white matter region and the corresponding estimated reference curve.

With the regional flow ratio measure *R*_1_ derived from [^15^O]water or [^11^C]PIB analysis for each voxel with a signal *n*(*t*), we simulated flow-adjusted reference curves (*n*_ND_(*t*)) for the corresponding voxel of the image, determining the tracer washout by the actual flow measured in the voxel. Here, the term *n*_ND_(*t*) represents the simulated dynamic wash-out of the tracer that *would have been* recorded from an individual voxel in the absence of binding. The result is a simulated image of the dynamic passages of the tracer through every voxel as functions of time in the absence of any binding in any voxel. The simulation is based on the dynamic behavior in a fixed reference region (cerebellar gray matter) given by *m*_ND_(*t*), which is the dynamic time-activity curve of the reference region. Figure 2 shows an example of the real and simulated non-displaceable (unbound) tracer time-activity curves for a small white matter region and a small putamen region, as well as the real time-activity curves for the ROIs. The obvious variation in the flow corrected reference curves illustrate that a single uncorrected reference curve may bias the result significantly. Further details can be found in the Theory section.

### Quantification of [^15^O]water accumulation as CBF (*K*^H_2_O^_1_)

The use of positron emission tomography with [^15^O]water in subjects included in the present analysis for the purpose of correction of measures of CBF for the effect of CO_2_ tension in blood were reported previously (Rodell et al., 2012). We quantified the CBF as the unidirectional blood-brain [^15^O]water clearance (*K*^H_2_O^_1_) in units of ml hg^−1^ min^−1^ with the linearized two-compartment model (Blomquist, 1984) modification of Ohta et al. (1996) and the Lawson–Hanson non-negative least squares solution to general least squares functions (Lawson and Hanson, 1974).

### Automatic detection of reference region

In order to validate the cerebellar gray matter as a non-specifically binding region, we mapped the distribution of the washout index Θ image, which has unit of time, as defined for the Hypotime method (Møller et al., 2009). Distinctive regions of high Θ values (i.e., close to T = Tomography duration) are indicative of a potential reference region, as previously demonstrated for the tracer [^11^C]WAY-100635 ([^11^C]WAY) (Hirvonen et al., 2007; Møller et al., 2009) where the cerebellar white matter served as the reference region.

## Results

### Wash-in and wash-out phases

Due to the rapid removal of the tracer from the circulation, analysis of the dynamic [^11^C]PIB record revealed an initial high frequency signal of arterial origin in the first 2 min of recording, followed first by a maximum peak (within the timeframe 2–10 min) from which there was only by washout. For the WARM method, we split the signal into the three time frames of, first, arterial phase, second, peak uptake, and third, wash-out. We used the maximum peak within (the 2–10 min peak uptake phase) for estimation of the relative uptake coefficient *R*^PIB^_1_ and the calibrated surrogate CBF index directly from the [^11^C]PIB signal. We used the wash-out phase from maximum peak within 2–60 min of the dynamic record for estimation of the binding potential *BP*_ND_.

### Signal-to-noise ratio and stability

**Figure 3:**
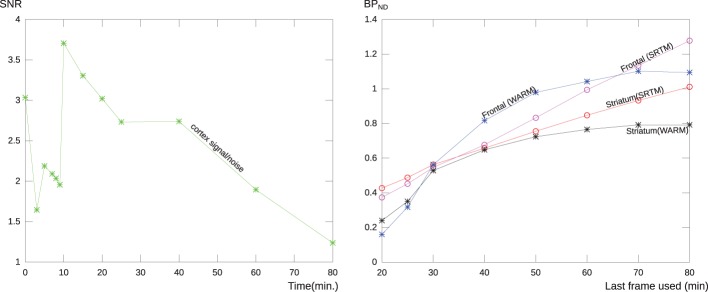
**The left panel shows the relative noise (*SNR*) of a typical PIB scan as a function of tomography time duration for a region covering the cerebral cortex without cerebellum**. After 60 min the noise level is more than half the signal, probably due to the loss of the signal due to decay of [^11^C] and washout of the tracer. the right panel shows the stability of the *BP*_ND_ measure for the WARM method and SRTM, for each method is shown the result of applying the analysis to images with different end frame times. The curves represent the parametric *BP*_ND_ values for a typical AD patient for a single voxel in frontal lobe and a striatal region mean value. As seen the methods are in relatively good agreement, but the WARM method converge with more and more data considered.

In order to establish the temporal dependence and stability of the WARM method in relation to tomography duration, we analyzed the performance of the WARM and SRTM methods at different scan-time. After 10 min, the signal-to-noise ratio increased for [^11^C]PIB because of the decay of the radioactivity and washout of the tracer. After 60 min, the standard deviation reached almost half the signal, as illustrated in Figure 3 (left panel). Figure 3 (right panel) shows the stability of the SRTM and WARM method results with respect to time in the tomograph. The WARM method results converge after 60 min, while the SRTM results retain some dependence on time. Considering the scale used for *BP*_ND_, the results of the two methods are in relative good agreement. Considering the SNR and stability together, we regard a maximum time of 60 min to be an acceptable compromise. For this reason, we confined the calculations of binding potential with the WARM method to the 2–60 min time window. The SRTM calculations were made with a maximum duration of 60 min.

### PIB retention

In order to test the methods' power to distinguish the AD from the HC subject groups by quantification of the amount of retention, we extracted regional parametric values for the PIB retention from the parametric images with the three different methods WARM, SRTM and SUVR. For the WARM, SRTM methods, we report mean absolute *BP*_ND_ values and for the SUVR method we extracted DVR values.

The mean absolute *BP*_ND_ values from the WARM and SRTM methods were in very good agreement for cerebral cortex values for the AD patients, while for the HC subjects the WARM method values are lower for most of cortex. The WARM method therefore yielded more significant differences between the AD and HC groups than the SRTM method for cortex (CORT), putamen (PU), frontal lobe (FL), occipital lobe (OL), temporal lobe (TL), and parietal lobe (PL). Only caudate nucleus (CN), white matter (WM) and cerebellum (CERB) reference values were less significant than with the SRTM method. Compared with the SUVR method, both the WARM and SRTM methods yielded greater between-group differences of retention. Table 1 lists the significance levels, and Table 2 lists the mean parametric estimates for each region and group.

**Table 1:** Regional statistical significance (student *t*-test) between AD vs. HC subject values for both [^11^C]PIB retention and flow estimates (^*^p < 0.05) (^**^p < 0.001).

**Region**	**PIB-retention**			**Flow**					
	**WARM**	**SRTM**	**SUVR**	**R^PIB^_1_**	**R_1_ (H_2_0)**	**CBF**	**CBF (pCO_2_ corrected)**	**PIB “CBF”**	**PIB “CBF” (pCO_2_ corrected)**
CORT	^*^0.0092	^*^0.0206	^*^0.0410	0.0366	0.0693	^*^0.0029	^**^0.0007	0.1933	0.2032
Pu	^*^0.0099	^*^0.0232	^*^0.0299	0.4069	0.4396	^*^0.0076	^*^ 0.0064	0.2582	0.3250
CN	^*^0.0161	^*^0.0078	0.4803	0.0848	0.1144	^*^0.0088	^**^0.0007	0.0762	0.0537
FL	^*^0.0139	^*^0.0282	^*^0.0379	0.0654	0.2033	^*^0.0045	^*^ 0.0016	0.2085	0.2343
OL	^*^0.0119	0.0600	0.2479	0.0771	0.0512	^*^0.0050	^*^0.0023	0.1834	0.2113
TL	^*^0.0026	^*^0.0170	^*^0.0228	^*^0.0065	^*^0.0099	^*^0.0016	^**^0.0001	0.1341	0.1165
PL	^*^0.0129	^*^0.0197	^*^0.0470	^*^0.0191	^*^0.0277	^*^0.0041	^**^0.0006	0.1323	0.1150
WM	^*^0.0241	^*^0.0146	0.7859	0.1006	0.1443	^*^0.0074	^*^0.0011	0.3473	0.4690
CERB	0.8383	0.3334	0.7517	0.3682	0.4561	^*^0.0190	^*^0.0196	0.6840	0.8808

**Table 2:** *BP*_ND_ and DVR values measured for AD vs. HC subjects using three different methods (^*^indicates statistical significance between subject groups *p* < 0.05).

**Region**	**WARM *BP*_ND_**		**SRTM *BP*_ND_**		**SUVR DVR**	
	**AD**	**HC**	**AD**	**HC**	**AD**	**HC**
CORT	^*^0.243 (0.146)	^*^0.083 (0.018)	^*^0.267 (0.125)	^*^0.140 (0.043)	^*^1.614 (0.460)	^*^1.209 (0.165)
Pu	^*^0.280 (0.163)	^*^0.101 (0.030)	^*^0.422 (0.139)	^*^0.270 (0.077)	^*^2.025 (0.512)	^*^1.517 (0.227)
CN	^*^0.282 (0.169)	^*^0.108 (0.041)	^*^0.339 (0.129)	^*^0.174 (0.055)	1.519 (0.499)	1.373 (0.229)
FL	^*^0.285 (0.192)	^*^0.090 (0.023)	^*^0.322 (0.175)	^*^0.156 (0.058)	^*^1.790 (0.607)	^*^1.248 (0.210)
OL	^*^0.180 (0.097)	^*^0.075 (0.021)	0.194 (0.88)	0.123 (0.033)	1.385 (0.360)	1.214 (0.147)
TL	^*^0.204 (0.096)	^*^0.074 (0.016)	^*^0.222 (0.80)	^*^0.129 (0.041)	^*^1.528 (0.365)	^*^1.144 (0.161)
PL	^*^0.281 (0.189)	^*^0.087 (0.020)	^*^0.299 (0.146)	^*^0.149 (0.049)	^*^1.835 (0.593)	^*^1.328 (0.217)
WM	^*^0.258 (0.073)	^*^0.173 (0.045)	^*^0.189 (0.065)	^*^0.116 (0.026)	1.990 (0.268)	1.954 (0.204)
CERB	0.107 (0.034)	0.111 (0.030)	0.111 (0.009)	0.103 (0.015)	0.981 (0.034)	0.987 (0.035)

Closer examination of the WM binding revealed a considerable amount of white matter retention with the WARM method, although the flow normalization accounted for some of this. The SRTM method yielded negative binding values for these WM areas (not visible within the specified range). We judge the negative values to be due to difficulties of regression to the WM dynamics.

**Figure 4:**
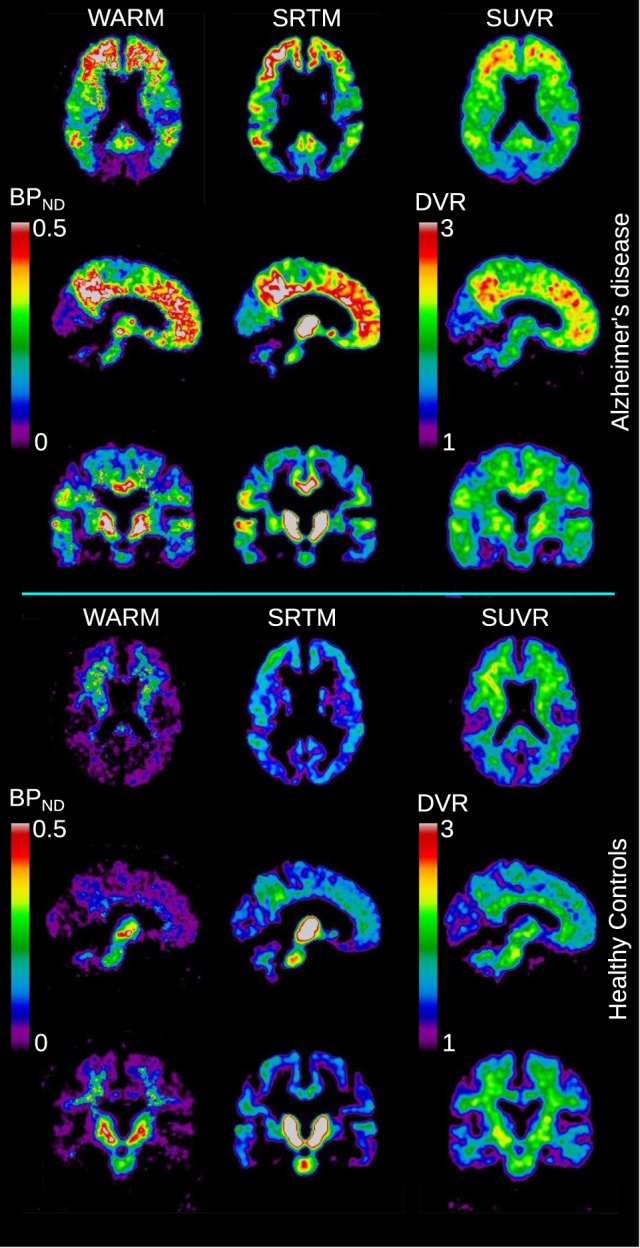
**Images of [^11^C]PIB binding in brain**. Upper three rows show mean binding images of sections of the brains of a group of AD patients (*N* = 6), and lower three rows show similar sections for age-matched healthy controls (*N* = 8). For each group is shown the *BP*_ND_ values derived from the WARM and SRTM methods. For the SUVR method, the mean DVR measures are illustrated in the range 1–3.

To illustrate the regional distribution of the binding, Figure 4 shows the group mean binding images for the three different methods. The WARM and SRTM method results are reported as *BP*_ND_ values, scaled similarly, while the SUVR values are reported as DVR values in the 1–3 range.

### Cerebral blood flow

Surprisingly, the absolute CBF values derived from [^15^O]water (Table 3) most significantly distinguished the AD from the HC groups (Table 1), especially after correction for the within-group variation of arterial *CO*_2_ tensions. The low standard deviations compared to the retention values probably accounted for the more significant differences among the CBF estimates. The CBF values of the reference region (cerebellar gray matter) differed among the groups. This difference disappeared when we considered the relative flow indices, i.e., the *R*_1_ values (Table 4). Thus, normalization may remove important differences and should be used with caution (Mayr, 1982; Borghammer et al., 2009a,b). The *R*_1_ estimates obtained with [^15^O]water and [^11^C]PIB were similar (Table 4), and for both methods the temporal and parietal lobes were the only regions with significantly different values when we compared AD and HC subjects. Hence, it is feasible to use *R*_1_ estimates from the [^11^C]PIB sequence to correct for intra-subject flow normalization, also when inter-individual flow differences may be of clinical value as a marker of AD pathology.

**Table 3:** **Regional absolute CBF measures before and after correction for PCO_2_ (Rodell et al., 2012)**.

**Region**	**CBF**		**CBF(pCO_2_ corrected)**	
	**AD ml hg^−1^ min^−1^**	**HC ml hg^−1^ min^−1^**	**AD ml hg^−1^ min^−1^**	**HC ml hg^−1^ min^−1^**
CORT	^*^40.14 (6.0)	^*^51.8 (5.0)	^**^37.6 (1.2)	54.4 (8.0)
Pu	53.8 (5.6)	65.5 (6.7)	^*^50.7 (3.9)	68.9 (11.6)
CN	^*^35.4 (6.0)	^*^47.5 (7.1)	^**^33.1 (1.4)	49.6 (7.8)
FL	^*^43.7 (6.6)	^*^54.4 (4.4)	^*^40.9 (2.0)	57.3 (8.5)
OL	^*^39.0 (4.8)	^*^50.3 (6.2)	^*^36.7 (3.4)	52.8 (8.6)
TL	^*^34.3 (7.4)	^*^48.4 (4.9)	^**^31.9 (2.0)	50.8 (7.1)
PL	^*^39.5 (8.4)	^*^54.0 (6.1)	^**^36.8 (3.7)	56.6 (8.7)
WM	^*^30.8 (4.7)	^*^39.1 (4.3)	^*^28.9 (1.4)	41.1 (6.0)
CERB	^*^46.9 (4.8)	^*^54.3 (4.7)	^*^44.3 (4.7)	57.2 (9.8)

*CBF values derived from the [^15^O]water images*.

* and

** indicates statistical significance between subject groups (^*^p < 0.05) (^**^p < 0.001).

**Table 4:** **Regional *R*_1_ measures relative to the cerebellar gray matter reference**.

	**R^PIB^_1_**		**R_1_H_2_O**	
**Region**	**AD**	**HC**	**AD**	**HC**
CORT	0.73 (0.05)	0.82 (0.08)	0.74 (0.06)	0.82 (0.07)
Pu	0.99 (0.06)	1.05 (0.13)	0.99 (0.06)	1.05 (0.14)
CN	0.65 (0.09)	0.76 (0.11)	0.65 (0.10)	0.76 (0.11)
FL	0.78 (0.07)	0.87 (0.07)	0.81 (0.09)	0.87 (0.07)
OL	0.72 (0.04)	0.80 (0.08)	0.72 (0.03)	0.80 (0.08)
TL	^*^0.62 (0.08)	^*^0.77 (0.07)	^*^0.63 (0.09)	^*^0.77 (0.07)
PL	^*^0.72 (0.09)	^*^0.86 (0.09)	^*^0.72 (0.10)	^*^0.86 (0.09)
WM	0.56 (0.04)	0.62 (0.07)	0.57 (0.05)	0.62 (0.07)
CERB	0.86 (0.2)	0.88 (0.03)	0.86 (0.02)	0.88 (0.03)

The measures were derived either from [^11^C]PIB images or from the [^15^O]water images (

* * indicates statistical significance between subject groups p < 0.05)*.

**Figure 5:**
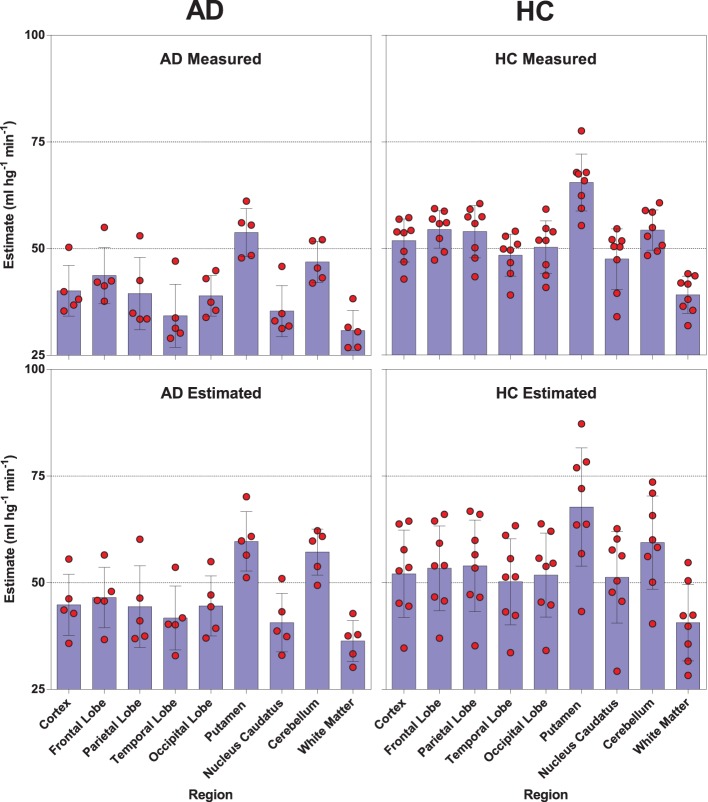
**Distribution and mean relationships between the [^11^C]PIB derived surrogate CBF values (lower panels) and the standard [^15^O]water derived CBF measures (upper panels) for selected regions in healthy controls (left panels) and AD patients (right panels)**.

**Figure 6:**
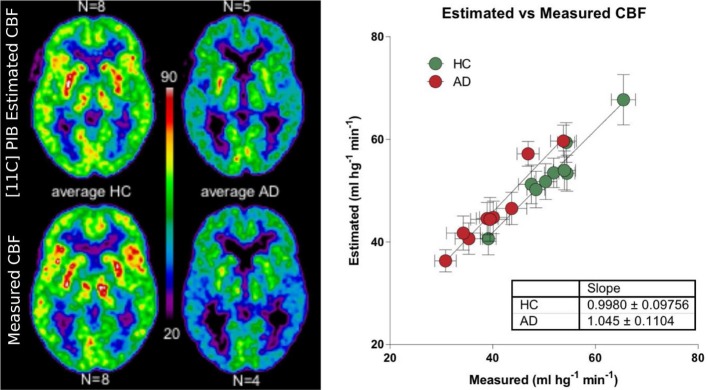
**Left panel shows relationship between the [^11^C]PIB estimated surrogate CBF measures and the standard [^15^O]water-measured CBF values for averages of healthy controls and AD patients (ml hg^−1^ min^−1^)**. Right panel shows the linear relationship between the different regions averaged for the HC and AD groups.

We used a site-specific scale factor to obtain a simulated absolute flow estimates from the PIB peak arrival characteristics, but this factor was derived only from healthy subjects. We did not find the same statistical differences for the simulated CBF values (Table 5) as for the standard CBF estimates. As illustrated for select regions in Figure 5, this failure may be due to the high variability observed in the simulated CBF values, as generally there was good correlation for the group means seen in Figure 6, except for white matter and cerebellar gray matter. Here, the simulated CBF values did not yield the same CBF value decrease for the AD subjects.

**Table 5:** **Regional surrogate CBF measures before and after correction for PCO_2_ (Rodell et al., 2012)**.

	**PIB “CBF”**		**PIB “CBF”(pCO_2_ corrected)**	
**Region**	**AD**	**HC**	**AD**	**HC**
CORT	44.8 (7.1)	52.1 (10.2)	44.9 (6.8)	49.8 (6.5)
Pu	59.7 (7.0)	67.7 (13.9)	59.9 (5.7)	64.9 (9.7)
CN	40.6 (6.8)	51.2 (10.7)	40.8 (6.1)	48.9 (6.9)
FL	46.5 (7.1)	53.3 (9.9)	46.7 (6.3)	51.2 (6.2)
OL	44.5 (7.1)	51.8 (9.9)	44.7 (6.3)	49.7 (6.6)
TL	41.7 (7.5)	50.2 (10.1)	41.8 (6.3)	48.1 (6.5)
PL	44.4 (9.6)	54.0 (10.7)	44.5 (8.3)	51.7 (6.8)
WM	36.3 (4.8)	40.6 (9.0)	36.5 (4.3)	39.0 (6.7)
CERB	57.2 (5.4)	59.3 (10.9)	57.5 (5.5)	56.9 (6.6)

*CBF values derived from the [^ıt 11^C]PIB images*.

### Reference region

**Figure 7:**
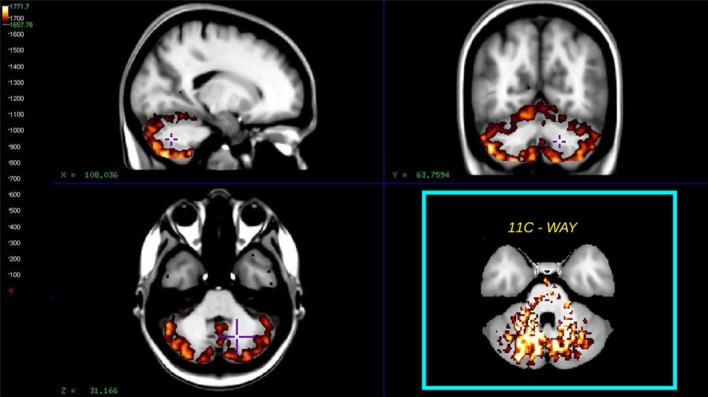
**The figure shows the average washout index Θ image calculated over the time interval *T* = 1890 s for all subjects**. Mean values >1660 s, superimposed on a non-linear registered average MRI brain, the cyan insert shows the similar reference region for the way compound as found in Møller et al. (2009).

A clear advantage of the Hypotime method was the production of washout indices in the form of Θ images with unit of time as an intermediate result (Møller et al., 2009). High Θ values (Θ > 1660 s relative to *T* = 1890 s) are indicative of the region's potential for service as reference. This was previously demonstrated in studies of the WAY tracer, where the reference region was selected as the cerebellar white matter. However, for the present tracer, the cerebellar gray matter consistently stood out as a reference region candidate. Figure 7 shows the average Θ washout index image for all subjects, for comparison with an insert of the reference region previously identified for WAY (Møller et al., 2009).

## Discussion

For [^11^C]PIB, the mono-exponential washout kinetics with rapid disappearance of tracer from the circulation complicates the qualitative assessment of binding with existing methods. Here we demonstrate the use of the novel flow-independent WARM to calculate the binding potential of [^11^C]PIB. The calculation is based on the clearance of the tracer from the brain, relative to the initial deposits in brain tissue, compared to the same measure in a non-binding reference region with the same initial deposit and washout characteristics of the cerebellar gray matter. We measure the binding either regionally by allometric linearization using Equation (41), or by direct parametric mapping using Equation (43). Compared to the standard SRTM and SUVR methods, WARM yielded binding measures with greater statistical discrimination between groups of AD and HC subjects for cortical gray-matter (excluding cerebellum), putamen, frontal, occipital, temporal, and parietal lobes, and lower differences for caudate nucleus, white matter, and especially cerebellum, as expected. This has relevance for the enhanced use of quantified [^11^C]PIB retention for clinical discrimination of AD, regardless of the relationship between amyloid-β deposits and the disease.

The WARM method revealed a converging stability of the *BP*_ND_ measure as a function of tomography duration with acceptable stability at 60 min, at a time when the SNR is still favorable. The method also takes advantage of direct calculation without regression. The direct calculation is important to its use because it serves to reduce tomography duration without compromise of the quantification, without the loss of the uptake signal, which we have shown to be useful for flow estimation.

Based on the Θ washout index with unit of time, defined in Møller et al. (2009), we found that the cerebellar gray matter consistently is the best choice of reference region with no specific binding of [^11^C]PIB. The striking difference between the cerebellar reference region for tracer WAY (Hirvonen et al., 2007; Møller et al., 2009) and [^11^C]PIB, testifies to the value of directly confirming the presence of a true reference area from the PET sequence.

We also showed the feasibility of using the initial *K*_1_ signal as a surrogate measure of absolute CBF, directly from the [^11^C]PIB image. However, compared to absolute CBF measures with [^15^O]water, the CBF index derived from [^11^C]PIB had greater variability. As [^11^C]PIB is comparatively lipophilic, there are probable permeability differences from [^15^O]water images (Gjedde et al., 2013), which may account for some of the variability. The absolute CBF estimates differed significantly for all regions, especially after correction for arterial PCO_2_ differences (Rodell et al., 2012), notably also for the cerebellar reference region. When we calculated the *R*_1_ values relative to the cerebellar reference, much of the difference was eliminated, and only parietal and temporal lobes had significant decline in the AD group. This observation shows that the normalization to relative flow measures may mask important disease specific information in AD, although it does enable the correction for the intra-individual flow bias (Borghammer et al., 2009a). The observation is of concern also to the MRI estimation of CBF indices which rarely are reported as calibrated measures. Clinically, the absolute CBF value should be regarded as an important imaging parameter for the diagnosis of AD, alongside the [^11^C]PIB retention, the fluorodeoxyglucose (FDG) uptake, and the accumulation of other markers.

Non-specific binding in white matter (WM) areas is found both when assessed *in vivo* by PET (Fodero-Tavoletti et al., 2009) and by postmortem autoradiography (Svedberg et al., 2009). This implies some non-specific binding to WM, also when tissue slices are superfused with the tracer, rather than accumulating the tracer from the circulation after transport across the blood-brain barrier. However, we find that the WM binding often observed with [^11^C]PIB is due both to slow washout kinetics and to non-specific retention that is not explicable by *R*_1_ differences.

In conclusion, we demonstrate that the WARM method yields a stable measure of PIB's binding potential with relative simplicity and reasonable tomography duration, employing only integration for noise reduction with no need for model regression. The washout index Θ is readily used to assess the suitability of a potential reference region. The method accounts for relative flow differences in the tissue and yields a calibrated measure of the absolute CBF, obtained directly from the [^11^C]PIB signal. Comparable with the SRTM and SUVR methods, the WARM method provides better discrimination between AD subjects and healthy controls. Taken together these characteristics (Table 6) merit further investigation of WARM for clinical use with [^11^C]PIB, and the method may be equally applicable to other washout tracers.

**Table 6:** **Characteristic feature of the three methods used**.

	**WARM**	**SRTM**	**SUVR**
Method approach	Calculation	Non-linear regression	Calculation
Simplicity	Moderate	Complex	Simple
R1 correction	Yes	Yes	No
Noise-reducing	Integration	Fitting	Steady-state average
Reported parameters	*BP*_ND_	*BP*_ND_	DVR
	*R*_1_	*R*_1_	
	“*CBF*”	*k*2	

## Theory

This section describes theoretical considerations regarding the three different approaches to the determination of the binding potential of [^11^C]PIB (*BP*_ND_) or DVR.

### Reference region ratio measure (SUVR)

The [^11^C]PIB retention can be calculated by determining the accumulation relative to a reference tissue to obtain a ratio measure (SUVR). The ratio measure is the fraction of the region-of-interest integral of [^11^C]PIB accumulation at steady-state, assumed to have been established no later than this time after injection (*t*_*s*_ = 40 min), extended to the end (*t*_*e*_ = 60 min). The volume of distribution of the tracer, *V*_T_ and the volume of distribution of non-displaceable tracer (*V*_ND_) define the binding potential *BP*_ND_.

(8) $$V_{\text{T}}=V_{\text{ND}}+BP_{\text{ND}}\;V_{\text{ND}}$$

(9) $$BP_{\text{ND}}\equiv \frac{V_{\text{T}}}{V_{\text{ND}}}-1$$

The signal measured by PET is the accumulated mass of the tracer (*V*_T_) relative to the integral of *c*_*a*_(*t*), the radioactivity of the tracer in the arterial circulation i.e.,

(10) $$V_{\text{T}}\equiv \frac{\int_{t_{s}}^{t_{e}}m(t)\text{ d}t}{\int_{t_{s}}^{t_{e}}c_{a}(t)\text{ d}t}$$

The variable of major interest to [^11^C]PIB binding to Aβ formations is the quantity of specific (i.e., displaceable) binding. This binding is expressed as the binding potential (*BP*_ND_), the displaceable tracer relative to the amount of non-displaceable tracer in the tissue. In any given region of interest (ROI), an area of reference may be referred to as a region where *equal* amounts of non-displaceable tracer enter and leave the tissue. Such a reference fulfills the two requirements that,

1. tracer enters the tissue in proportion to blood flow,
2. unbound tracer (i.e., tracer dissolved in *V*_ND_) clears the tissue at the rate determined by the blood flow, as defined for a tracer subject to flow-limited exchange across the blood-brain barrier.

For the reference region, *V*_T_ ≡ *V*_ND_, and Equation (10) defines the steady-state volume of distribution,

(11) $$V_{\text{ND}}\equiv \frac{\int_{t_{s}}^{t_{e}}m_{\text{ND}}(t)\text{ d}t}{\int_{t_{s}}^{t_{e}}c_{a}(t)\text{ d}t}$$

As the total amount of radioactivity in the arterial blood are the same in the two regions, i.e., the ROI (Equation 10), and the reference region (Equation 11), the binding potential is obtained from the Equation (9).

(12) $$BP_{\text{ND}}=\frac{\int_{t_{s}}^{t_{e}}m(t)\text{ d}t}{\int_{t_{s}}^{t_{e}}m_{\text{ND}}(t)\text{ d}t}-1$$

Equation (12) is valid when requirements (1) and (2) are fulfilled. However, for a tracer that disappears rapidly from the blood stream, (1) is invalid after the initial wave of tracer has passed the tissue. When the concentrations *c*_*a*_(*t*) in the circulation primarily depend on the wash-out and hence mainly on the CBF. Requirement (2) depends on the regional CBF and is valid for tracers subject to flow-limited exchange with brain tissue.

It is improbable that we would find a reference region for all tissues and tracers that uphold these requirements, unless additional tomography is completed with a similar tracer of no specific binding and hence no displacement. However, when we identify a single homogeneous reference region for [^11^C]PIB, we can mimic the tracer's behavior as reference for any given ROI.

### Initial [^11^C]PIB distribution as surrogate CBF measure

The two factors that affect the accumulation of [^11^C]PIB after the initial distribution are the magnitude of blood flow, which mediates washout, and the degree of binding, which limits the rate of washout. In order to distinguish these factors and hence to assess the binding of [^11^C]PIB in any given region or voxel, we determine the relative flow ratios *R*_1_ as the magnitude of CBF in a region or voxel, relative to CBF in the reference region, defined as *R*_1_ = CBF/CBF_ND_.

Cerebral blood flow can be assessed in two different ways, either directly by means of PET with [^15^O]water (CBF), or a surrogate measure (CBF^PIB^) approximated indirectly from the [^11^C]PIB signal at the peak of distribution. This approximation is made by first excluding the instantaneous arterial distribution in the first 2 min after injection. In the subsequent 8 min, the initial distribution of the [^11^C]PIB signal depends largely on the wash-in. Until maximum peak values are reached at Δ*t*_*p*_, the wash-in can be expressed as the unidirectional clearance (*K*_1_) from the blood into the brain when no tracer has left the brain yet.

We recently determined the permeability-surface product (PS) for [^11^C]PIB (Gjedde et al., 2013). There the *K*_1_ measure from the initial distribution of the [^11^C]PIB signal is related to the CBF by the Renkin–Crone formula (Crone, 1963; Renkin, 1964),

(13) $$K_{1}=\text{CBF}\left(1-e^{-\frac{\text{PS}}{\text{CBF}}}\right)$$

where by definition the extraction fraction E from blood into tissue is,

(14) $$E=\left(1-e^{-\frac{\text{PS}}{\text{CBF}}}\right)$$

From Equation (13), it follows that the wash-in ratio

(15) $$R_{1}^{\text{PIB}}=K_{1}/K_{1}^{\text{ND}}$$

relative to a reference region is linked to the similar ratio for blood flow *R*_1_ = CBF/CBF_ND_ by the relative extraction fraction relative to the extraction fraction of the reference region *E*_ND_

(16) $$R_{1}^{\text{PIB}}=R_{1}\frac{E}{E_{\text{ND}}}.$$

This relation indicates that the *R*^PIB^_1_ approximates *R*_1_ for extraction fractions similar to the reference region, which is the case for compounds with sufficiently high PS products and for regions where the CBF is close to the CBF_ND_ of the reference region. With these limitations, and for want of known CBF values, *R*^PIB^_1_ may be estimated from the maximum signal intensity value *m*(Δ*t*_*p*_), such that *R*^PIB^_1_ = m(Δ*t*_*p*_)/*m*_ND_(Δ*t*^ref^_*p*_), relative to the maximum signal intensity of the reference region at time Δ*t*^ref^_*p*_.

A tentative absolute measure for *K*_1_ can be estimated by additionally accounting for differences in the delay of the maximum peak and by normalizing for weight and dose, (*m*(Δ*t*_*p*_)/Δ*t*_*p*_) * Δ*t*^ref^_*p*_ * weight/dose provides an delay normalized estimate of the magnitude of *K*_1_. The estimate of *K*_1_ is scaled by a site- and tomograph-specific constant *K*_site_ for cerebral cortex values in order to derive tentative absolute flow estimates CBF^PIB^ from the [^11^C]PIB images. This derivation requires calibration to a normal material of estimates of cortex *K*_1_ and CBF values, with tight adherence to the protocol used in the calibration.

### Flow dependence of specific binding measure

With the regional flow ratio measure *R*_1_ derived from [^15^O]water or [^11^C]PIB analysis for each voxel with a signal *n*(*t*), we simulated flow-adjusted reference curves (*n*_ND_(*t*)) for the corresponding voxel of the image, determining the tracer washout by the actual flow measured in the voxel. Here, the term *n*_ND_(*t*) represents the simulated dynamic wash-out of the tracer that *would have been* recorded from an individual voxel in the absence of binding. The result is a simulated image of the dynamic passages of the tracer through every voxel as functions of time in the absence of any binding in any voxel. The simulation is based on the dynamic behavior in a fixed reference region (cerebellar gray matter) given by *m*_ND_(*t*), which is the dynamic time-activity curve of the reference region.

In order to determine the simulated reference curve *n*_ND_(*t*) from the measured reference curve *m*_ND_(*t*), let a non-binding voxel or region have a flow CBF_*n*_, and let it refer to a measured region with flow CBF_ND_. Each region has washout rates given by *k*_2_ = CBF_*n*_/*V*_ND_ and *k*^ND^_2_ = CBF_ND_/*V*_ND_, respectively. Then *R*_1_ relates to the variables as indicated by the following equations,

(17) $$R_{1}\equiv \frac{n(0)}{m_{\text{ND}}(0)}=\frac{k_{2}}{k_{2}^{\text{ND}}}=\frac{{\text{CBF}}_{n}}{{\text{CBF}}_{\text{ND}}}$$

where each voxel is the site of mono-exponential washout given by rate constants of the magnitudes,

(18) $$k_{2}^{\text{ND}}=-\frac{1}{t}\ln\left(\frac{m_{\text{ND}}(t)}{m_{\text{ND}}(0)}\right)$$

and

(19) $$k_{2}=-\frac{1}{t}\ln\left(\frac{n_{\text{ND}}(t)}{n_{\text{ND}}(0)}\right)$$

where *R*_1_ relates the *k*_2_ terms as,

(20) $$k_{2}=-\frac{R_{1}}{t}\ln\left(\frac{m_{\text{ND}}(t)}{m_{\text{ND}}(0)}\right)$$

such that Equations (19) and (20) yield *n*_ND_(*t*) as,

(21) $$\ln\left(\frac{n_{\text{ND}}(t)}{n_{\text{ND}}(0)}\right)=R_{1}\ln\left(\frac{m_{\text{ND}}(t)}{m_{\text{ND}}(0)}\right)$$

where

(22) $$n_{\text{ND}}(t)=n_{\text{ND}}(0)e^{R_{1}\ln\left(\frac{m_{\text{ND}}(t)}{m_{\text{ND}}(0)}\right)}$$

or,

(23) $$n_{\text{ND}}(t)=n_{\text{ND}}(0){\left[\frac{m_{\text{ND}}(t)}{m_{\text{ND}}(0)}\right]}^{R_{1}}$$

where *n*_ND_(*t*) is the simulated flow-adjusted reference curve corresponding to the measured curve n(*t*) when equal amounts of tracer enter, i.e., *n*(0) = *n*_ND_(0). When expressed for every voxel, the procedure yields a new dynamic reference image *n*_ND_(*t*) that enables the comparisons among the flow-involved SUVR method and the flow-devolved WARM and SRTM methods, as the flow-devolved version of Equation (12) can now be expressed as,

(24) $$BP_{\text{ND}}=\frac{\int_{t_{s}}^{t_{e}}n(t)\text{ d}t}{\int_{t_{s}}^{t_{e}}n_{\text{ND}}(t)\text{ d}t}-1$$

Figure 2 shows an example of the real and simulated non-displaceable (unbound) tracer time-activity curves for a small white matter region and a small putamen region, as well as the real time-activity curves for the ROIs. The variation in the simulated flow corrected reference curves, illustrate that a single uncorrected reference curve may bias the result significantly and may only be valid for regions where the flow is equal to in this case the cerebellum.

### Washout allometric reference method (WARM)

In the case of negligible input from the circulation after the initial brief uptake, the differential equations (25) and (26)

(25) $$\frac{\text{d}m^{*}(t)}{\text{d}t}=K_{1}c_{a}(t)-k_{2a}m^{*}(t)$$

and

(26) $$\frac{\text{d}m_{\text{ND}}^{*}(t)}{\text{d}t}=K_{1}^{\text{ND}}c_{a}(t)-k_{2}^{\text{ND}}m_{\text{ND}}^{*}(t)$$

are linked only while the tracer is dispersed from well-defined *c*_*a*_, i.e., during the brief uptake period until maximum peak (within 2–10 min timeframe) when washout is assumed to be negligible.

The term *K*_1_ is the unidirectional clearance of the tracer *c*_*a*_ by the tissue, *K*^ND^_1_ is the clearance of the tracer *c*_*a*_ by the reference region, m^*^ and m^*^_ND_ are the measured PET signal in the tissue (with displaceable binding) and reference, respectively. The term *k*_2*a*_ defines the apparent measurable washout rate constant for the ROI. The term *k*_2_ is the unknown washout rate for non-specifically bound tracer of the same region of interest, and *k*^ND^_2_ defines the measurable washout rate of non-specifically bound tracer in the reference tissue into the plasma. The uncoupling of the first and the second term on the right hand side of the equations means that elimination of the first *K*_1_ and *K*^ND^_1_ terms yields the equations.

(27) $$\frac{{\text{dm}}^{*}(\text{t})}{\text{dt}}=-k_{2a}m^{*}(t)$$

(28) $$\frac{{\text{dm}}_{\text{ND}}^{*}(\text{t})}{\text{dt}}=-k_{2}^{\text{ND}}m_{\text{ND}}^{*}(t)$$

This defines the first-order decay for 1-compartment first order kinetics, assuming that association and dissociation is sufficiently rapid in the tissue compartments. The Equations (27) and (28) each predict a mono-exponential washout from the time *t*_0_ where the [^11^C]PIB signal depends only on the washout rate. The total volume of distribution in the binding region then is simplified to (Lammertsma and Hume, 1996; Møller et al., 2009)

(29) $$V_{\text{T}}=\frac{K_{1}}{k_{2a}}=\frac{K_{1}\left(1+BP_{\text{ND}}\right)}{k_{2}}$$

where

(30) $$k_{2a}=\frac{k_{2}}{\left(1+BP_{\text{ND}}\right)}$$

As the differential Equations (25) and (26) are linked in the initial phase (described by *K*_1_), the ratio *R*_1_ = *K*_1_/*K*^ND^_1_ accounts for the difference of delivery to the regions of interest and reference. For the non-specifically bound tracer, we assume that the volume of distribution is the same in all regions, i.e.,

(31) $$V_{\text{ND}}=\frac{K_{1}}{k_{2}}=\frac{K_{1}^{\text{ND}}}{k_{2}^{\text{ND}}}$$

Consequently the rate constants *k*_2_ and *k*^ND^_2_ of washout of non-specifically bound tracer from tissue to plasma are similarly linked by *R*_1_,

(32) $$k_{2}=R_{1}k_{2}^{\text{ND}}$$

by combining which Equations (30) and (32) BPND is found as

(33) $$BP_{\text{ND}}=R_{1}\left(\frac{k_{2}^{\text{ND}}}{k_{2a}}\right)-1$$

The late uncoupled mono-exponential differential equations (27) and (28) can be rearranged to

(34) $$\frac{\text{d}m^{*}(t)}{m^{*}(t)}=-k_{2a}\text{d}t$$

(35) $$\frac{\text{d}m_{\text{ND}}^{*}(t)}{m_{\text{ND}}^{*}(t)}=-k_{2}^{\text{ND}}\text{d}t$$

Integrating both sides the gives

(36) $$\ln\left(\frac{m^{*}(t)}{m^{*}(0)}\right)=-k_{2a}t$$

(37) $$\ln\left(\frac{m_{\text{ND}}^{*}(t)}{m_{\text{ND}}^{*}(0)}\right)=-k_{2}^{\text{ND}}t$$

where m^*^(0) = m^*^(*t*_0_) and m^*^_ND_(0) = m^*^_ND_(*t*_0_) are the initial value at start of the washout *t*_0_ = 2 min. By dividing Equation (37) by (36)

(38) $$\frac{\ln\left(\frac{m_{\text{ND}}^{*}(t)}{m_{\text{ND}}^{*}(0)}\right)}{\ln\left(\frac{m^{*}(t)}{m^{*}(0)}\right)}=\frac{k_{2}^{\text{ND}}t}{k_{2a}t}$$

The right hand side of this equation can be expressed in terms of the binding potential by use of Equation (33).

(39) $$\frac{\ln\left(\frac{m_{\text{ND}}^{*}(t)}{m_{\text{ND}}^{*}(0)}\right)}{\ln\left(\frac{m^{*}(t)}{m^{*}(0)}\right)}=\frac{\left(1+BP_{ND}\right)}{R_{1}}$$

where (1 + *BP*_ND_) is the distribution volume ratio(DVR). Alternatively written

(40) $$BP_{\text{ND}}+1=\frac{R_{1}\ln\left(\frac{m_{\text{ND}}^{*}(t)}{m_{\text{ND}}^{*}(0)}\right)}{\ln\left(\frac{m^{*}(t)}{m^{*}(0)}\right)}=\frac{m^{*}(0)\ln\left(\frac{m_{\text{ND}}^{*}(t)}{m_{\text{ND}}^{*}(0)}\right)}{m_{\text{ND}}^{*}(0)\ln\left(\frac{m^{*}(t)}{m^{*}(0)}\right)};$$

where (1 + *BP*_ND_) is the distribution volume ratio(DVR).

Equation (39) can also be expressed as an allometric relationship between the logarithmic of the fraction of remaining f tracer in a ROI and the reference region where $\left(\frac{DVR}{R_{1}}\right)$ is the scaling exponent, found in this formulation by log–log linearization as,

(41) $$\ln\left(\frac{m_{\text{ND}}^{*}(t)}{m_{\text{ND}}^{*}(0)}\right)=\left(\frac{DVR}{R_{1}}\right)\ln\left(\frac{m^{*}(t)}{m^{*}(0)}\right)$$

For direct calculation without linearization, Equation (39) was expressed in terms of integrations and differences of the logarithms, assuming constant *BP*_ND_ and *R*_1_, which greatly reduces noise in *BP*_ND_ estimates.

(42) $$BP_{\text{ND}}(T)=\frac{R_{1}\int_{0}^{T}(\ln\left(m_{\text{ND}}^{*}(t)\right)-\ln\left(m_{\text{ND}}^{*}(0)\right)\text{ d}t}{\int_{0}^{T}\left(\ln\left(m^{*}(t)\right)-\ln\left(m^{*}(0)\right)\right)\text{ d}t}-1$$

That we further simplified with Equation (17) to the operational equation,

(43) $$BP_{\text{ND}}(T)=\frac{m^{*}(0)\int_{0}^{T}\left(\ln\left(m_{\text{ND}}^{*}(t)\right)-\ln\left(m_{\text{ND}}^{*}(0)\right)\right)\text{ d}t}{m_{\text{ND}}^{*}(0)\int_{0}^{T}\left(\ln\left(m^{*}(t)\right)-\ln\left(m^{*}(0)\right)\right)\text{ d}t}-1$$

When log transformed, the fraction (i.e., DVR) part of this equations states that the nominator is the accumulated log-signal for the reference tissue relative to how much was present before washout, this difference is scaled by the initial tracer amount of the ROI. The denominator describes the accumulated log-signal for a ROI or voxel relative to how much was present before washout. This difference is scaled by the start amount of the reference region. Thus the fraction is corrected both for flow, i.e., initially deposited tracer, and the exponential behavior of the washout. Figure 1 illustrates the behavior of the nominator, denominator and *BP*_ND_(*T*) of Equations (43) and (40) for simulated ROI and reference curves with 20% added Gaussian noise. As seen the nominator and denominator from Equation (40) (in panel 2 from the left) are stabilized by the integration in Equation (43) (panel 3 from the left). Panel 4 illustrates how the *BP*_ND_(*T*) values converge towards the theoretical result.

### Simplified reference tissue method

The simplified reference tissue method (SRTM) (Lammertsma and Hume, 1996) yields binding potential when a single tissue compartment model fits the data. SRTM solves differential equations similar to (25) and (26) (equations (1) and (5) in the paper of Lammertsma and Hume (1996)). The method assumes that these differential equations are coupled by tracer distribution in the circulation throughout the entire duration of the tomography.

### Conflict of interest statement

The authors declare that the research was conducted in the absence of any commercial or financial relationships that could be construed as a potential conflict of interest.
